# Association of Tumor Size With Myometrial Invasion, Lymphovascular Space Invasion, Lymph Node Metastasis, and Recurrence in Endometrial Cancer: A Meta-Analysis of 40 Studies With 53,276 Patients

**DOI:** 10.3389/fonc.2022.881850

**Published:** 2022-06-02

**Authors:** Xiaoying Jin, Chunjuan Shen, Xiaodi Yang, Yayuan Yu, Jianzhang Wang, Xuan Che

**Affiliations:** ^1^ Department of Obstetrics and Gynecology, Jiaxing University Affiliated Maternity and Child Hospital, Jiaxing, China; ^2^ Department of Gynecology, Women’s Hospital, School of Medicine, Zhejiang University, Hangzhou, China

**Keywords:** endometrial cancer, tumor size, myometrial invasion, lymphovascular space invasion, lymph node metastasis, recurrence, overall survival

## Abstract

**Background:**

Myometrial invasion (MI), lymphovascular space invasion (LVSI), and lymph node metastasis (LNM) have been found to have independent prognostic factors in endometrial cancer. Tumor size has practical advantages in endometrial cancer. The cutoff values for tumor size conformed with current literature. More and more studies inferred that tumor size >20 mm showed a strong correlation. However, the relationship between tumor size >20 mm and MI, LVSI, LNM, recurrence, and overall survival (OS) remains controversial, and no meta-analysis has been conducted. Therefore, a systematic review and meta-analysis should be performed to discuss this issue later on.

**Methods:**

Relevant articles were collected from PubMed, EMBASE, and Cochrane Library databases from January 1990 to June 2021. The predictive value of tumor size >20 mm in endometrial cancer was studied, and data were pooled for meta-analysis using Review Manager 5.1. Additionally, the odds ratio (OR) was analyzed, and cumulative analyses of hazard ratio (HR) and their corresponding 95% CI were conducted.

**Results:**

A total of 40 articles with 53,276 endometrial cancer patients were included in the meta-analysis. It contained 7 articles for MI, 6 for LVSI, 21 for LNM, 7 for recurrence, and 3 for OS. Primary tumor size >20 mm was significantly associated with depth of MI (OR = 5.59, 95% CI [5.02, 6.23], *p* < 0.001), positive LVSI (OR = 3.35, 95% CI [2.34, 4.78], *p* < 0.001), positive LNM (OR = 4.11, 95% CI [3.63, 4.66], *p* < 0.001), and recurrence (OR = 3.52, 95% CI [2.39, 5.19], *p* < 0.001). Tumor size >20 mm was also related to OS *via* meta-synthesis of HR in univariate survival (HR 2.13, 95% CI [1.28, 3.53], *p* = 0.003). There was no significant publication bias in this study by funnel plot analysis.

**Conclusion:**

Primary tumor size >20 mm was an independent predictive factor for the depth of MI, positive LVSI, positive LNM, recurrence, and poor OS. Therefore, it is more important to take into account the value of tumor size in the clinicopathological staging of endometrial carcinoma. Tumor size >20 mm should be integrated into the intraoperative algorithm for performing a full surgical staging. Well-designed and multicenter studies, with a larger sample size, are still required to verify the findings.

## Introduction

Endometrial cancer is the sixth most common neoplasm in women worldwide, and the incidence rate is increasing rapidly (1). The International Federation of Gynecology and Obstetrics (FIGO) mandated that the treatment of endometrial cancer was surgical staging, which includes hysterectomy, bilateral salpingo-oophorectomy, or pelvic and para-aortic lymphadenectomy (2). A gynecologic oncology group study identified some risk factors, such as stage, histological subtype, depth of myometrial invasion (MI), lymphovascular space invasion (LVSI), grade, and lymph node metastasis (LNM), which could predict recurrence and survival (3).

A gynecologic oncology group study in 1987 proposed that primary tumor size was not considered a risk factor for lymphatic metastasis (4). Some published studies indicated tumor size was not a risk associated with recurrence in women with endometrial cancer (5, 6). However, other literature showed that tumor size seemed to be a significant risk factor for endometrial cancer (7, 8). Recent data suggested that primary tumor size was an important parameter in predicting the clinicopathological outcomes for endometrial cancer patients, but it seemed to be controversial. Gusberg et al. firstly implied that it came out to be a poor prognosis with a tumor size of >10 cm (9). Riggs et al. analyzed the optimal tumor diameter that can predict LNM and was noted to be 35 mm (10). The Mayo Criteria, which included the FIGO grade 1 or 2 endometrioid cancer, with tumor size <20 mm, MI < 50%, and no intraoperative evidence of macroscopic disease, was used to guide lymphadenectomy assessment (11). Milwaukee Model suggested that primary tumor size >50 mm and MI > 33% identifies possible lymphatic dissemination in low-risk endometrial cancer patients (12). The cutoff values for tumor size conformed with current literature, which varies from 20 to 50 mm (12, 13). Kilt et al. explored that cutoff of tumor size increasing from 20 to 30 and 50 mm had a lower at-risk rate of lymph node dissection but an unacceptably high false-negative rate (14). Tumor sizes <20 mm for low-risk endometrial cancer remained more sensitive than those with tumor sizes <30 mm for identifying lymphatic dissemination (14). Recently, more and more studies inferred that a tumor size of 20 mm remains clinically significant in relation to the risk of recurrence (7, 8). Therefore, we should focus on the relationship between the tumor size of 20 mm and MI, LVSI, LNM, recurrence, and OS.

There was no meta-analysis about the relationship between tumor size >20 mm and MI, LVSI, LNM, recurrence, and OS. The aim of our study was to investigate the relationship between primary tumor size of 20 mm and clinicopathological parameters, recurrence, and OS.

## Methods

### Literature Search Strategy

A rigorous search of the PubMed, EMBASE, and Cochrane Library databases from January 1990 to June 2021 was undertaken to identify relevant articles. The key search terms were drafted as follows: “tumor size,” “tumor diameter,” “uterine cancer,” “uterine carcinoma,” “endometrial cancer,” “endometrial carcinoma,” “prognosis,” “prognostic factor,” “risk,” “myometrial invasion,” “lymphovascular space invasion,” “lymph node metastasis,” “recurrence,” and “overall survival.” The literature search was performed by two authors independently.

### Criteria for Inclusion and Exclusion

The inclusion criteria included the following: 1) the patients were only diagnosed with endometrial cancer; 2) tumor size, which was defined as a cutoff of 20 mm; 3) one or more main clinicopathological factors included MI, LVSI, LNM, recurrence, and OS; and 4) article was published in English. The exclusion criteria included the following terms: 1) letters, editorials, expert opinions, reviews, and animal studies; 2) preoperative tumor size at MRI and PET/CT or ultrasound; and 3) studies of data were insufficient.

### Data Extraction

The data from the selected trials were extracted and assessed by two authors independently. Any disagreements in data extraction were resolved by further discussion and consensus. Three categories of data extraction in each study are the following: baseline patient characteristics, clinicopathological outcomes, and survival outcomes. Baseline characteristics of the included studies need the first author’s name, study publication year, country, and sample size. Clinicopathological outcomes included MI, LVSI, and LNM. Survival outcomes included recurrence and OS.

### Data Analysis

All statistical analyses were performed by using the Cochrane Collaboration’s Review Manager Software 5.1. Clinicopathological outcomes and recurrence were pooled as odds ratio (OR) and 95% CI. Pooled hazard ratio (HR) and corresponding 95% CI were used to analyze the association between tumor size and OS. Fixed- or random-effects meta-analysis models were varied according to the existence of heterogeneity among the included studies. It appeared that heterogeneity with chi-square *p* > 0.1 and/or I^2^ > 50%, publication bias was evaluated by the shape of the funnel plot. The test for funnel plot asymmetry was applied only when at least 10 studies were included in a meta-analysis. A significant statistical difference was pointed out when a *p*-value was less than 0.05.

The quality of the included studies was assessed by the Quality Assessment of Diagnostic Accuracy Studies-2 (QUADAS-2), which is essential to evaluate the risk of bias for included studies.

## Results

### Study Characteristics

**Figure 1:**
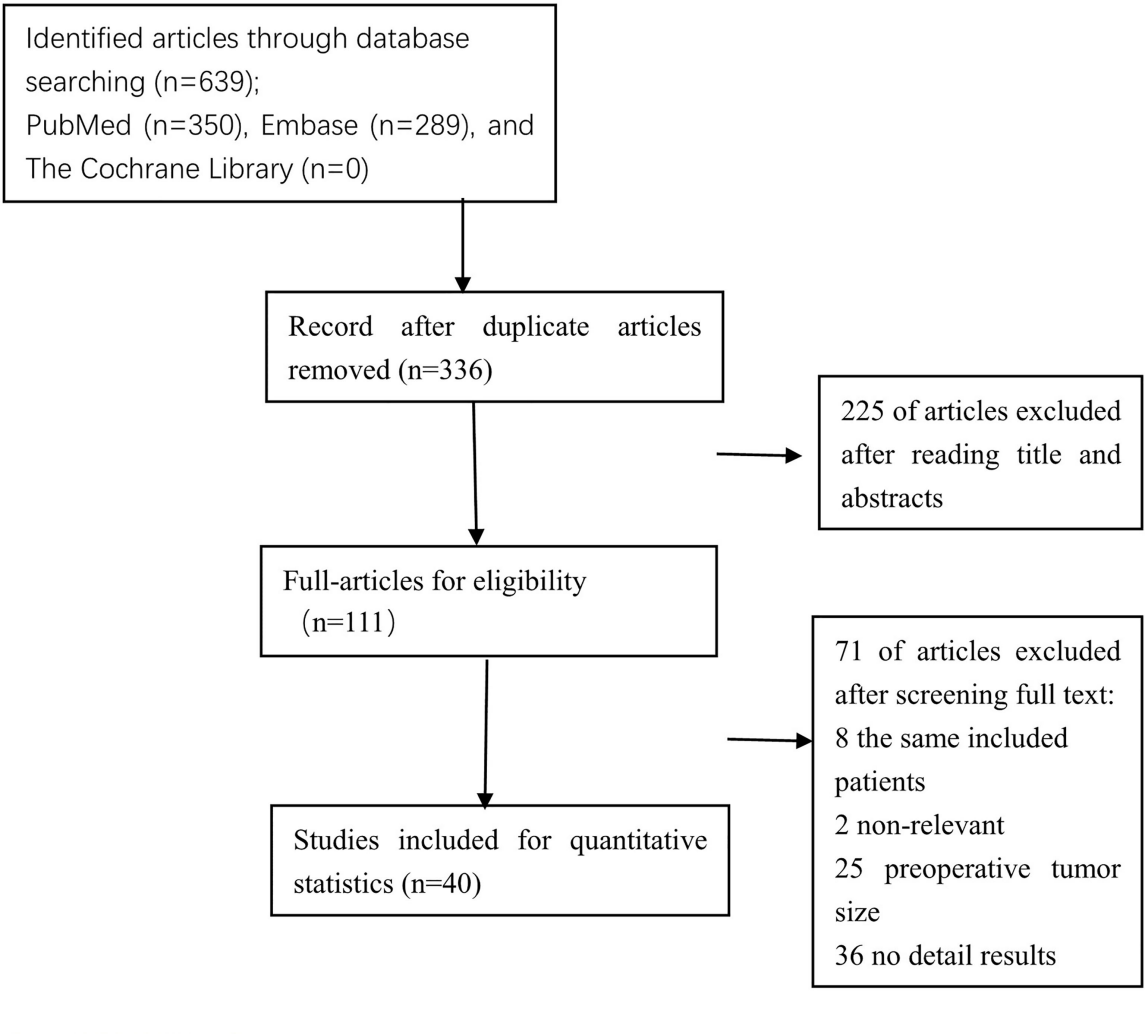
Preferred Reporting Items for Systematic Reviews and Meta-Analyses (PRISMA) flow diagram.

The Preferred Reporting Items for Systematic Reviews and Meta-Analyses (PRISMA) flow diagram was shown in **Figure 1**. After titles and abstracts were screened, 225 records were excluded, including 97 that indicated that the cutoff tumor size was not 20 mm, 100 that indicated the preoperative tumor size, 21 without original data, and 7 without relevant outcome. A full text of 111 articles was assessed, 71 records were excluded, including studies with the same included patients, 2 that indicated HRs from univariate survival analyses not available, 25 that indicated preoperative tumor size, 36 that have no detailed results, and finally, forty studies with a total of 53,276 eligible patients. Baseline characteristics of the included studies are shown in **Table 1**. All of the included studies were retrospectively designed, including 7 for MI (20, 24, 27, 35, 44, 45, 48), 6 for LVSI (20, 35, 41–44), 27 for LNM (15–40, 45), 7 for recurrence (5–8, 20, 46, 47), and 3 for OS (16, 49, 50). Included studies consisted of 2 large-scale retrospective cohort studies (27, 38). The results of the meta-analysis are summarized in **Table 2**.

**Table 1:** Baseline characteristics of the included studies.

First author	Year	Country	n	Stage	Tumor Grade	Histologic	Risk factors
Akıs (15)	2021	Turkey	146	I–III	I–III	Endometrioid	LNM
AlHilli (16)	2013	USA	883	I–IV	I–III	Endometrioid	LNM OS
Boyraz (17)	2017	Turkey	191	IA	I–II	Endometrioid	LNM
Boyraz (18)	2018	Turkey	307	NA	I–III	Endometrioid	LNM
Chang (19)	2011	Korea	203	I–IV	I–III	Mixed	Paraaortic LNM
Doll (20)	2014	USA	183	I–IV	High	Mixed	LNM LVSI Recurrence MI
Dali (21)	2019	USA	58	I	NA	Endometrioid	LNM
Gilani (22)	2014	USA	207	NA	I–III	Endometrioid	LNM
Günakan (23)	2019	Turkey	762	I–IV	I–III	Mixed	LNM
Karalok (24)	2017	Turkey	368	NA	I–III	Endometrioid	LNM MI
Lee (25)	2009	Korea	834	I–IV	I–III	Endometrioid	LNM
Li (26)	2019	China	874	I–III	I–III	Mixed	LNM
Mahdi (27)	2014	USA	19692	I	I–III	Endometrioid	LNM MI
Matsushita (28)	2019	Japan	185	I–IV	I–III	Endometrioid	LNM
Milam (29)	2012	USA	971	II–III	II–III	Endometrioid	LNM
Oz (30)	2017	Turkey	243	I	I	Endometrioid	LNM
Pavlakis (31)	2017	Greece	290	I–II	I	Endometrioid	LNM
Rathod (32)	2014	India	52	I–III	I–III	Mixed	LNM
Sari (33)	2017	Turkey	641	I–IV	I–III	Mixed	LNM
Shah (34)	2005	USA	194	I–IV	I–III	Mixed	LNM
Tecellioglu (35)	2021	Turkey	100	I–IV	I–III	Endometrioid	LVSI LNM MI
Turan (36)	2011	Turkey	198	I–IV	I–III	Mixed	LNM
Vaizoglu (37)	2013	Turkey	261	I	I–III	Endometrioid	Retroperitonea LNM
Vargas (38)	2014	USA	21011	NA	I–III	Endometrioid	LNM
Watanabe (39)	2003	Japan	107	I–III	I–II	Endometrioid	Pelvic LNM
Zanfagnin (40)	2019	USA	83	IIIC	I–III	Mixed	Pelvic LNM
Ilker (41)	2015	Turkey	47	I–III	II–III	Mixed	LVSI
Oliver-Perez (42)	2021	Spain	220	I–III	I–III	Mixed	LVSI
Ayhan (43)	2018	Turkey	912	I–IV	I–II	Endometrioid	LVSI
Laufer (44)	2013	Italy	181	I	I–III	Endometrioid	LVSI MI
Schink (45)	1991	Chicago	125	NA	I–III	Mixed	MI LNM
Gadducci (6)	2009	Italy	32	I–II	I–III	Endometrioid	Recurrence
Bendifallah (46)	2014	France	396	I–III	I–III	Mixed	Recurrence
Güngördük (7)	2018	Turkey	279	IA	I–II	Endometrioid	Recurrence
ÇAKIR (47)	2019	Turkey	550	I–II	I–III	Endometrioid	Recurrence
Nwachukwu (5)	2021	Japan	222	IA	I	Endometrioid	Recurrence
LiMingzhu (8)	2014	China	398	I–II	NA	Endometrioid	Recurrence
Marcickiewicz (48)	2010	Sweden	214	I–IV	I–III	Mixed	MI
Roma (49)	2015	USA	589	NA	I–II	Endometrioid	OS
Yamada (50)	2020	Japan	67	I–IV	I–III	Mixed	OS

OS, overall survival; LVSI, lymphovascular space invasion; MI, myometrial invasion.

**Table 2:** The results of meta-analysis.

Analysis	Subgroup	Number of studies	Heterogeneity	Pooled result
			*χ^2^ I^2^ P*	OR/HR(95% CI) P
Tumor size and MI	In all FIGO stages	7	10.93 45% 0.09	5.59 (5.02–6.23) <0.001
Tumor size and LVSI	In all FIGO stages	6	4.55 0% 0.47	3.35 (2.34–4.78) <0.001
Tumor size and LNM	*In all FIGO stages*	27	20.28 0% 0.73	4.11 (3.63–4.66) <0.001
	In FIGO stage I–II	6	1.38 0% 0.85	3.69 (2.97–4.60) <0.001
	In all FIGO stages excluding I–II	21	18.12 0% 0.58	4.32 (3.71–5.03) <0.001
Tumor size and recurrence	In all FIGO stages	7	4.16 0% 0.66	3.52 (2.39–5.19) <0.001
	In FIGO stage IA	2	0.32 0% 0.57	5.94 (2.83–12.44) <0.001
	In FIGO stage I–II	3	0.72 0% 0.70	3.15 (1.72–5.78) <0.001
	In FIGO stage I–III	3	0.09 0% 0.77	2.37 (1.18–4.77) <0.001
Tumor size and overall survival	In all FIGO stages	3	7.79 61% 0.05	2.13 (1.28–3.53)* 0.003

^*^HR (95% CI).

### Literature Quality

**Figure 2:**
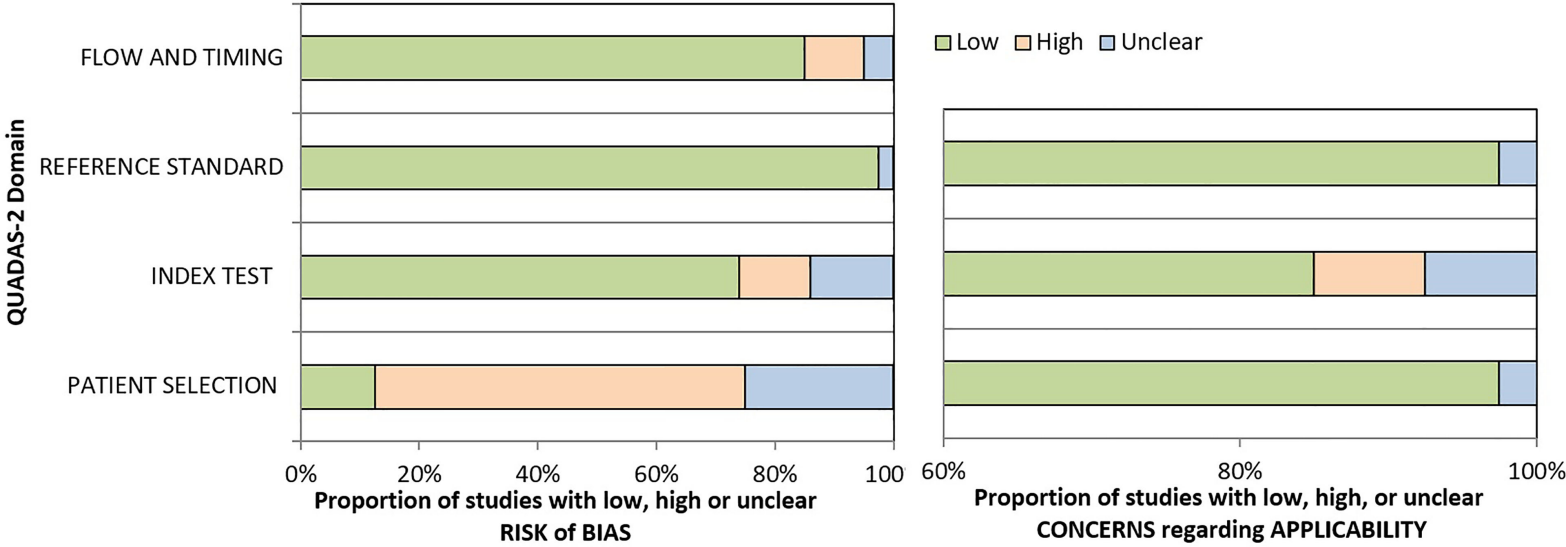
Quality Assessment of Diagnostic Accuracy Studies-2.

The QUADAS-2 was used to evaluate the quality of the included studies. Two reviewers independently evaluated the quality of the included 40 studies. The outcome is shown in **Figure 2**.

### Correlation Between Tumor Size and Myometrial Invasion in Endometrial Cancer

**Figure 3:**
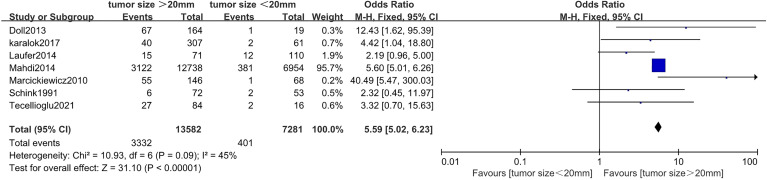
Forest plots showing the correlation between tumor size and myometrial invasion ( > 50%).

Seven studies (20, 24, 27, 35, 44, 45, 48) including 20,863 endometrial cancer patients were eligible to analyze the association between tumor size and MI in endometrial cancer. Pooled analysis showed that tumor size >20 mm was significantly associated with incidences of depth of MI (>50%) (OR = 5.59, 95% CI [5.02, 6.23], *p* < 0.001, I^2^ = 45%, *p* = 0.09) (**Figure 3**).

### Correlation Between Tumor Size and Lymphovascular Space Invasion in Endometrial Cancer

**Figure 4:**
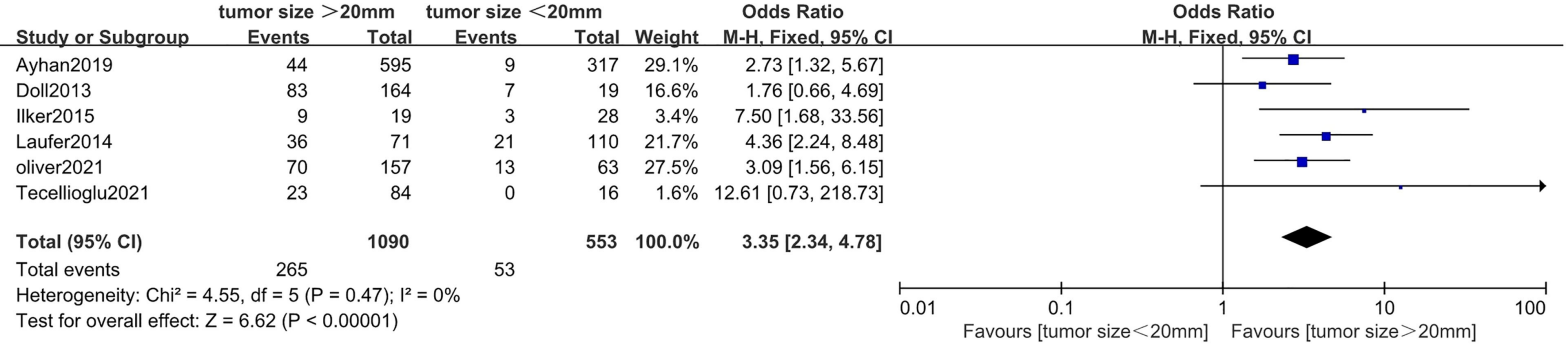
Forest plots showing the correlation between tumor size and lymphovascular space invasion (LVSI).

Six studies (20, 35, 41–44) with a total of 1,643 endometrial cancer patients were included for this analysis. The results of the pooled analysis revealed that tumor size >20 mm was significantly associated with positive LVSI (OR = 3.35, 95% CI [2.34, 4.78], *p* < 0.001, I^2^ = 0%, *p* = 0.47) (**Figure 4**).

### Correlation Between Tumor Size and Lymph Node Metastasis in Endometrial Cancer

**Figure 5:**
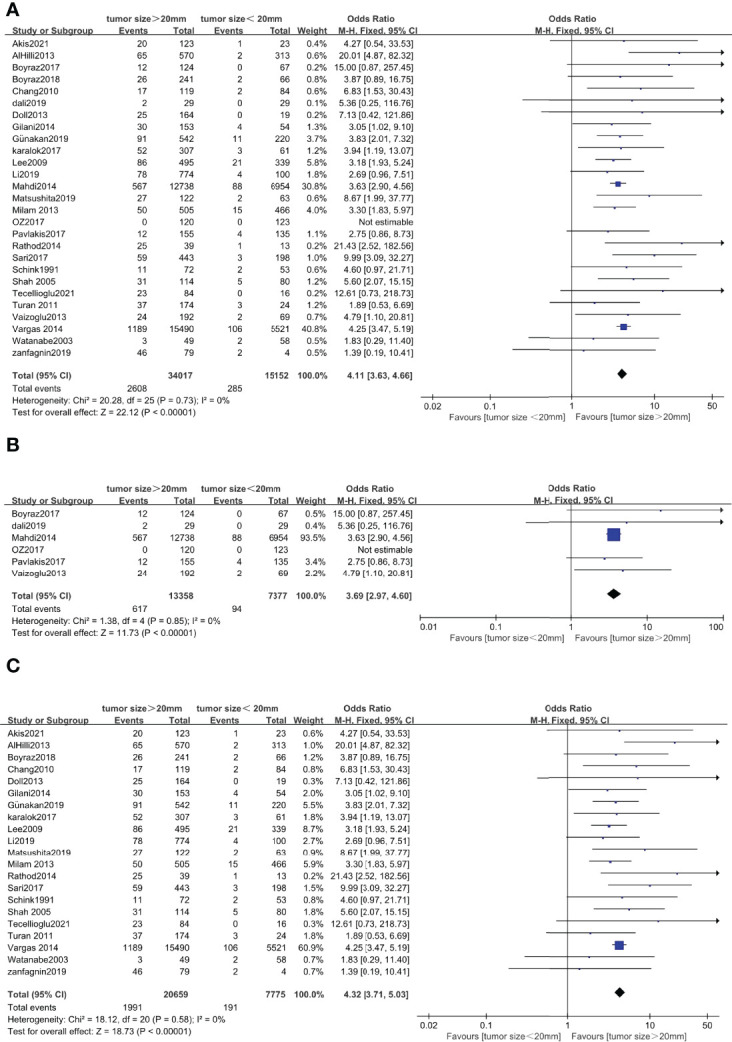
Forest plots showing the correlation between tumor size and lymph node metastasis (LNM). **(A)** All International Federation of Gynecology and Obstetrics (FIGO) stages. **(B)** FIGO stage I–II. **(C)** FIGO stage I–IV excluding stage I–II.

Twenty-seven studies with a total of 49,169 endometrial cancer patients were presented on the debate of association between tumor size and LNM (15–40, 45). The results of the pooled analysis revealed that tumor size >20 mm was significantly associated with LNM (OR = 4.11, 95% CI [3.63, 4.66], *p* < 0.001, I^2^ = 0%, *p* = 0.73). A total of 20,735 patients in FIGO stage I–II endometrial cancer that were based on 6 studies (17, 21, 27, 30, 31, 37) were enrolled in our meta-analysis. The pooled result showed that tumor size >20 mm was correlated with high LNM, and the pooled OR was 3.69 (95% CI [2.97, 4.60], *p* < 0.001), with heterogeneity (I^2^ = 0%, *p* = 0.85). A total of 28,434 patients had FIGO stage III–IV endometrial cancer, based on 21 studies that were enrolled in our meta-analysis (15, 16, 18–20, 22–26, 28, 29, 32–36, 38–40, 45). The pooled result showed that tumor size >20 mm was correlated with high LNM, and the pooled OR was 4.32 (95% CI [3.71, 5.03], *p* < 0.001), with heterogeneity (I^2^ = 0%, *p* = 0.58) (**Figure 5**).

### Correlation Between Tumor Size and Recurrence in Endometrial Cancer

**Figure 6:**
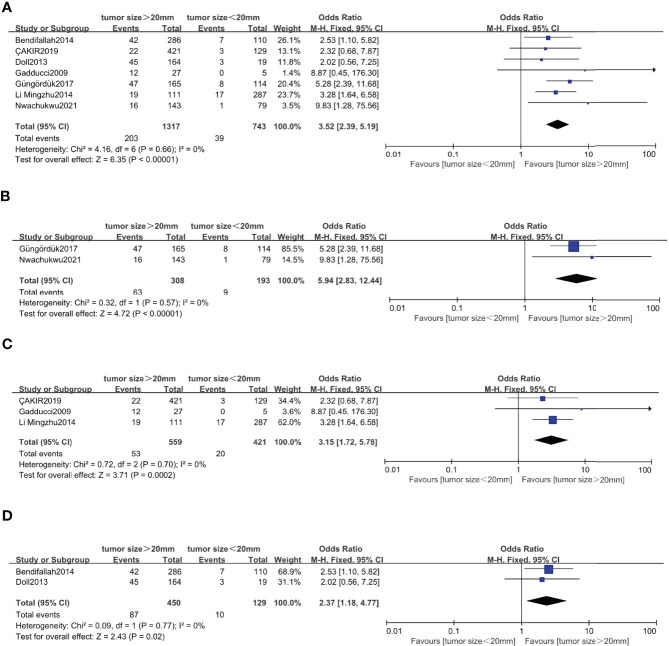
Forest plots showing the correlation between tumor size and recurrence. **(A)** All International Federation of Gynecology and Obstetrics (FIGO) stage. **(B)** FIGO IA. **(C)** FIGO stage I–II. **(D)** FIGO stage I–III.

Seven studies (5–8, 20, 46, 47) with a total of 2,060 endometrial cancer patients were eligible for analysis of the association between tumor size and recurrence. The pooled analysis revealed that tumor size >20 mm was significantly associated with recurrence (OR = 3.52, 95% CI [2.39, 5.19], *p* < 0.001, I^2^ = 0%, *p* = 0.66). A total of 501 patients in FIGO stage IA endometrial cancer, based on 2 studies, were enrolled in our meta-analysis (5, 7). The pooled result showed that tumor size >20 mm was correlated with high recurrence, and the pooled OR was 5.94 (95% CI [2.83, 12.44], *p* < 0.001), with heterogeneity (I^2^ = 0%, *p* = 0.57). A total of 980 patients in FIGO stage I–II endometrial cancer, based on 3 studies, were enrolled in our meta-analysis (6, 8, 47). The pooled result showed that tumor size >20 mm was also correlated with high recurrence, and OR was 3.15 (95% CI [1.72, 5.78], *p* < 0.001), with heterogeneity (I^2^ = 0%, *p* = 0.70). A total of 579 patients in FIGO stage I–III endometrial cancer, based on 2 studies, were enrolled in our meta-analysis (20, 46). The pooled result showed that tumor size >20 mm was also correlated with high recurrence, and OR was 2.37 (95% CI [1.18, 4.77], *p* < 0.001), with heterogeneity (I^2^ = 0%, *p* = 0.77) (**Figure 6**).

### Correlation Between Tumor Size and Overall Survival in Endometrial Cancer

**Figure 7:**
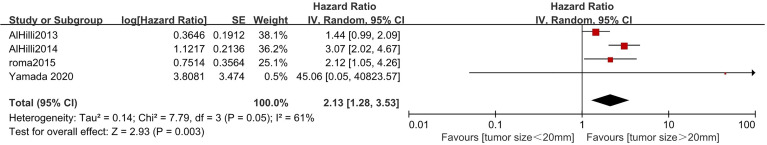
Meta-analysis of the association between tumor size and overall survival in endometrial cancer patients according to hazard ratio (HR) from univariate survival analyses.

Three studies (16, 49, 50) with a total number of 1,937 endometrial cancer patients were presented on the debate of tumor size >20 mm and OS. The random-effects model was applied for the significant heterogeneity. The pooled HRs of OS for univariate analyses were 2.13 (95% CI [1.28, 3.53], *p* = 0.003), with heterogeneity (I^2^ = 61%, *p* = 0.05) (**Figure 7**).

### Publication Bias of Included Studies

**Figure 8:**
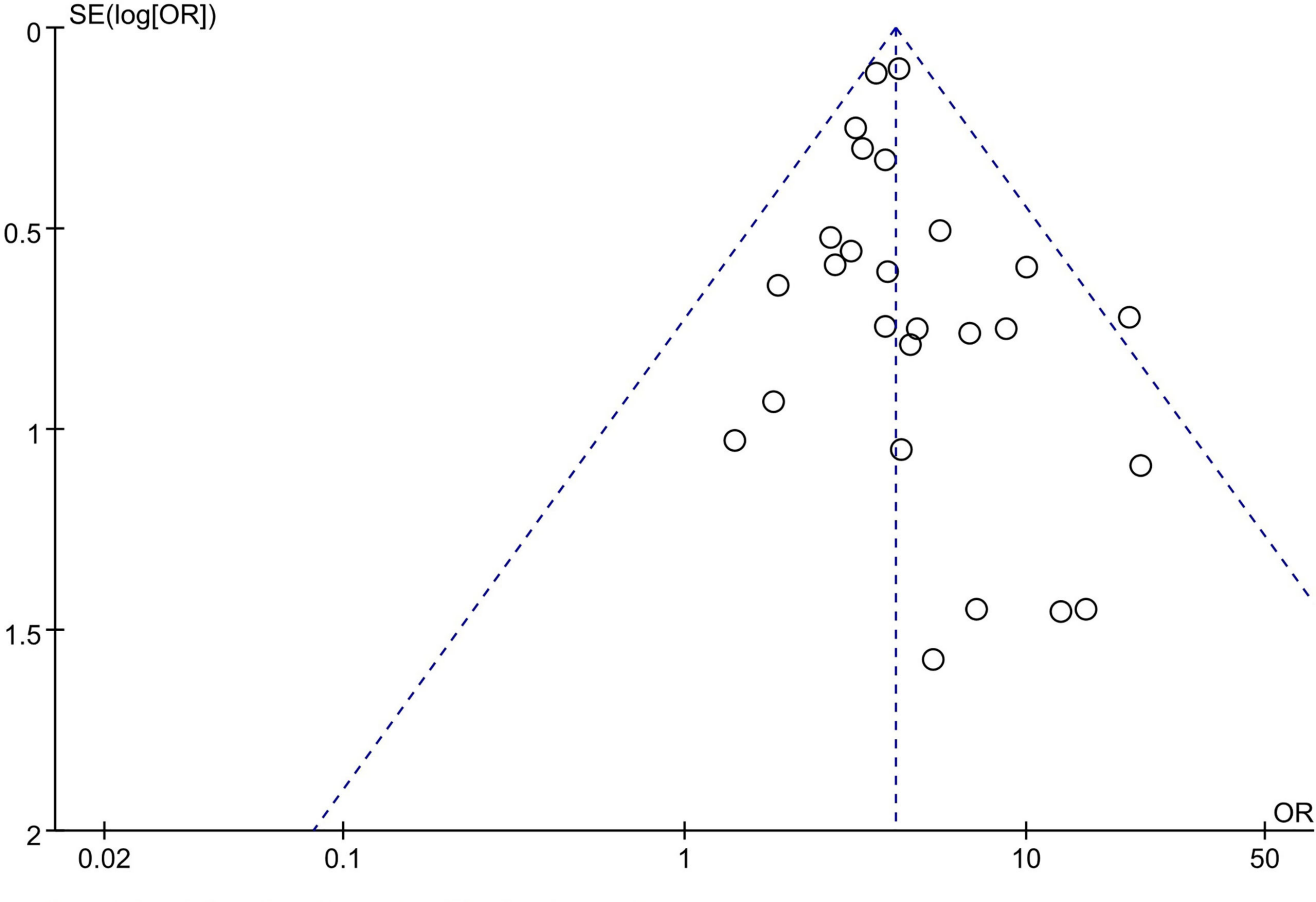
Funnel plot analysis of tumor size and lymph node metastasis.

A funnel plot was applied for the assessment of publication bias in the literature. The funnel plot for the included 27 studies on the association between tumor size and LNM was relatively symmetrical. Thus, there was no significant publication bias risk in all included studies investigating the association between tumor size and LNM (**Figure 8**).

## Discussion

A few published studies indicated that tumor size >20 mm could provide important prognostic outcomes for endometrial cancer (27, 45, 51, 52), but others showed that tumor size of 20 mm was not a prognostic factor in endometrial cancer (20, 47). In the current study, we performed a meta-analysis to roundly evaluate the prognostic value of tumor size. Our conclusion showed tumor size >20 mm was characterized by the presence of MI, which has 50% of patients with all FIGO stages in endometrial cancer. MI is vitally important in the development of endometrial cancer and a well-recognized predictor of extra-uterine spread (4, 53). MI is quite an early action of cancer cells, which classifies patients with initial stages as low-risk or high-risk patients for surgical planning (53). Depth of MI (>50%) definitely correlated to LVSI, LNM, recurrence, and OS (53).

Six studies with a total of 1,643 endometrial cancer patients were eligible for analysis, and the results demonstrated that tumor size >20 mm has a significant prognostic implication for positive LVSI. A retrospective analysis reported the impact on positive LVSI was more relevant than MI > 50% for predicting survival in stage I endometrial cancer (43). Positive LVSI should be emphasized in early-stage endometrial cancer (54). Moreover, these as well as other studies substantiated the fact that positive LVSI patients had lower recurrence-free survival and OS rates (55). The European Society of Gynaecological Oncology (ESGO) guidelines introduced that positive LVSI should recommend lymphadenectomy (56). Unfortunately, it is usually not possible to diagnose LVSI status on the frozen section, until the final pathology report. So tumor size may be a useful tool for predicting markers of LVSI in a preoperative or intraoperative surgical stage.

We have reached an agreement that LNM was one of the most important prognostic factors. Lymphadenectomy is the most component of the surgical procedure, providing survival benefits in the early stages of endometrial cancer (57). However, it could increase morbidity and postoperative complications (58). Yet it is important to emphasize that there is usually a more difficult procedure to readily evaluate MI, LVSI, and LNM on a frozen diagnosis. Thus, it is liable to measure tumor diameter macroscopically. In addition, it is more feasible to measure the tumor size before surgery. Our pooling data have shown that tumor size >20 mm was significantly correlated with higher incidences of LNM, whether in surgically FIGO stage I or FIGO stage I–IV. Based on our results, tumor size from intraoperative and preoperative could plan the surgery strategy, which may minimize the risk of complications, lower the burden of operation, and decrease morbidity or mortality.

Han et al. investigated different prognostic factors for the recurrence in stage IA and IB endometrial cancer. MI was the prognostic factor in stage IA, whereas the grade was the prognostic factor in stage IB (59). Our findings disclosed that the prevalence of tumor size >20 mm increased the risk of recurrence in FIGO IA endometrial cancer. We also found out that tumor size >20 mm significantly predicted higher recurrence in FIGO I–II/I–III endometrial cancer. Multivariate analysis showed that LVSI and depth of MI were independent risks for recurrence (49). Our pooled analysis also showed that tumor size >20 mm was a risk associated with LVSI and depth of MI, as well as higher recurrence. As it turned out, tumor size >20 mm was related to a greater risk of OS based on univariate survival analysis. Furthermore, we discovered that tumor size >20 mm could predict poorer OS in endometrial cancer.

Currently, gynecologists usually do not attach great importance to tumor size. In the evaluation criteria for the surgical–pathological staging, treatment, and prognosis of endometrial cancer, tumor size was rarely covered, and thereby its role may be underestimated. The relationship between tumor size and MI, LVSI, LNM, recurrence, and OS remains controversial. Therefore, we conducted this meta-analysis to investigate the relationship between primary tumor size of 20 mm and clinicopathological parameters, recurrence, and OS. The results showed that tumor size >20 mm was an independent predictive factor for the depth of MI, positive LVSI, positive LNM, recurrence, and poor OS, indicating the importance of tumor size. Tumor size >20 mm may provide additional information before surgery. Therefore, it is more important to take into account the value of tumor size in the clinicopathological staging of endometrial carcinoma.

The strength of the study was the first meta-analysis to discuss the value of tumor size >20 mm to predict clinicopathological outcomes and recurrence in patients with endometrial cancer. Nonetheless, the limitations of this meta-analysis included retrospective and non-randomized studies. In addition, the different cutoffs of tumor size will directly affect the association with the outcome. Other tumor sizes were not studied in the meta-analysis. A standardized cutoff of tumor size for future trials and studies should be highlighted.

## Conclusion

The meta-analysis showed that tumor size >20 mm was an independent predictive factor for the depth of MI, positive LVSI, positive LNM, recurrence, and poor OS, indicating the importance of tumor size in endometrial cancer. Therefore, it is more important to take into account the value of tumor size in the clinicopathological staging of endometrial carcinoma. Tumor size >20 mm should be integrated into the intraoperative algorithm for performing a full surgical staging.

## Data Availability Statement

The original contributions presented in the study are included in the article/supplementary material. Further inquiries can be directed to the corresponding authors.

## Author Contributions

CS and XJ contributed equally to this work. CS and XJ: conceptualization, literature retrieval, data acquisition, and writing of the manuscript. XY and YY: statistical analysis. JW and XC: manuscript review and editing. All authors contributed to the article and approved the submitted version.

## Funding

This study was supported by the National Natural Science Foundation of China (Grant numbers: 81802591 and 81974225) and the Science and Technology Bureau of Jiaxing city (Grant number: 2021AY30004).

## Conflict of Interest

The authors declare that the research was conducted in the absence of any commercial or financial relationships that could be construed as a potential conflict of interest.

## Publisher’s Note

All claims expressed in this article are solely those of the authors and do not necessarily represent those of their affiliated organizations, or those of the publisher, the editors and the reviewers. Any product that may be evaluated in this article, or claim that may be made by its manufacturer, is not guaranteed or endorsed by the publisher.
